# Reply to Çınar et al. Comment on “Zamfir et al. Hematologic Malignancies Diagnosed in the Context of the mRNA COVID-19 Vaccination Campaign: A Report of Two Cases. *Medicina* 2022, *58*, 874”

**DOI:** 10.3390/medicina58111576

**Published:** 2022-11-01

**Authors:** Ana Caruntu, Constantin Caruntu, Cristian Scheau

**Affiliations:** 1 Department of Oral and Maxillofacial Surgery, “Carol Davila” Central Military Emergency Hospital, 010825 Bucharest, Romania; 2 Department of Oral and Maxillofacial Surgery, Faculty of Dental Medicine, “Titu Maiorescu” University, 031593 Bucharest, Romania; 3 Department of Physiology, “Carol Davila” University of Medicine and Pharmacy, 050474 Bucharest, Romania; 4 Department of Dermatology, “Prof. N.C. Paulescu” National Institute of Diabetes, Nutrition and Metabolic Diseases, 011233 Bucharest, Romania

We would like to thank Çınar et al. for their appreciation and insightful discussions presented in their comment [1] on our recently published paper [2].

The COVID-19 pandemic has brought together medical specialists from multiple fields in a global effort to develop an efficient vaccine. Through tremendous efforts and close collaboration, specific messenger RNA (mRNA) vaccines were quickly developed and offered to the population through intense vaccination campaigns around the world. This was possible due to research and technological advancements, as well as ample, coordinated funding efforts. The scientific and editorial community continued the work by evaluating the efficacy and safety of the mRNA vaccines, constantly providing scientific data reported from worldwide studies.

From the beginning, the COVID-19 outbreak has challenged the management of patients diagnosed with hematological malignancies, due to the complex interconnections between an unknown viral infection and a dysfunctional immune system. After the development of specific vaccines, especially through the novel mRNA technology, another concern was the potentially negative effects of vaccination due to the stimulation of the immune response, especially in populations with pre-existing disorders of the immune system. Currently, it is known that the COVID-19 infection can yield significant hematological complications and affect the clinical evolution of hematological malignancies [3]. Attempts to identify methods for better management of patients with hematological malignancies and COVID-19 infection have provided favorable results [4]. A very encouraging recent report by Borgogna et al. has shown that patients with hematological malignancies that have recovered from COVID-19 can develop a robust humoral response after vaccination [5]. Protective levels of antibodies were, therefore, achieved with one vaccine dose in previously infected patients.

In their paper, Çınar et al. [1] share the experience of their reference hematological center and present four cases of acute myeloid leukemia diagnosed at different time intervals after the administration of the COVID-19 mRNA BNT162b2 vaccine. An interesting aspect of their case series is the heterogeneous clinical exhibition of the disease, as well as the diversity of vaccination regimens administered to these patients. Once more we emphasize that both our reports present only a chronological link between the inoculation of mRNA vaccines and the occurrence of different types of hematological malignancies. To our knowledge, until now there are only a few publications that have reported recurrence or exacerbation of hematological malignancies in patients that underwent mRNA vaccines for COVID-19 [6,7]. We share the general point of view that it is not possible to demonstrate direct causality between the vaccine and these types of adverse effects at this point. A recent meta-analysis has shown the vaccines to be safe to use in patients with hematological malignancies [8], albeit immunocompromised patients with lymphoid malignancies or undergoing hematopoietic cell transplantation or anti-CD20 antibody therapy may need supplementary doses to achieve adequate levels of spike subunit 1 serum immunoglobulin G [9,10,11].

In response to the comment of Çınar and colleagues [1], we agree with their observation that the COVID-19 vaccines have saved the world from the calamitous effects of the SARS-CoV-2 virus, and cumulative efforts should continue to prevent more disruptions, complications, and deaths. Additionally, as recent data suggests, patients with hematologic malignancies present higher risks than the normal population as they can develop persistent COVID-19 syndrome, have higher mortality and complication rates, and vaccination may reduce these risks [12]. However, pharmacovigilance is required to thoroughly monitor all clinical events occurring after novel pharmaceutical products. Specifically for preventive medication, such as mRNA vaccines, which exert their beneficial effects through immune mechanisms, it is important to monitor, report and examine all types of events related to the immune system, in order to identify, investigate and address accordingly potential side effects, for the general benefit of the population.

Furthermore, we agree with the comment of Çınar et al. [1] that continuous efforts should be made to study and accurately depict the mechanisms of action and the long-term pathophysiological implications of mRNA vaccines. The study team of our article is a strong advocate for evidence-based medicine, and in this perspective, the safety and efficacy of mRNA vaccines have definitely been proven, through extensive pre-clinical and clinical studies. In the meantime, we adhere to pharmacovigilance principles, and from this perspective, an open and, at the same time, judicious approach towards potential side effects should generate a detailed investigation, which in the end will complete the knowledge regarding novel pharmaceutical products and increase the general trust in medicine.

## References

[1] Çınar O.E., Erdoğdu B., Karadeniz M., Ünal S., Malkan Ü.Y., Göker H., Haznedaroğlu İ.C. (2022). Comment on Zamfir et al. Hematologic Malignancies Diagnosed in the Context of the mRNA COVID-19 Vaccination Campaign: A Report of Two Cases. *Medicina* 2022, *58*, 874. Medicina.

[2] Zamfir M.-A., Moraru L., Dobrea C., Scheau A.-E., Iacob S., Moldovan C., Scheau C., Caruntu C., Caruntu A. (2022). Hematologic Malignancies Diagnosed in the Context of the mRNA COVID-19 Vaccination Campaign: A Report of Two Cases. Medicina.

[3] Malkan U.Y., Haznedaroglu I.C. (2022). Hematological aspects of the COVID-19 syndrome. Eur. Rev. Med. Pharmacol. Sci..

[4] Visco C., Marcheselli L., Mina R., Sassone M., Guidetti A., Penna D., Cattaneo C., Bonuomo V., Busca A., Ferreri A.J.M. (2022). A prognostic model for patients with lymphoma and COVID-19: A multicentre cohort study. Blood Adv..

[5] Borgogna C., Bruna R., Griffante G., Martuscelli L., De Andrea M., Ferrante D., Patriarca A., Mahmoud A.M., Ucciero M.A.M., Gaidano V. (2022). Induction of robust humoral immunity against SARS-CoV-2 after vaccine administration in previously infected haematological cancer patients. Br. J. Haematol..

[6] Brumfiel C.M., Patel M.H., DiCaudo D.J., Rosenthal A.C., Pittelkow M.R., Mangold A.R. (2021). Recurrence of primary cutaneous CD30-positive lymphoproliferative disorder following COVID-19 vaccination. Leuk. Lymphoma.

[7] Goldman S., Bron D., Tousseyn T., Vierasu I., Dewispelaere L., Heimann P., Cogan E., Goldman M. (2021). Rapid Progression of Angioimmunoblastic T Cell Lymphoma Following BNT162b2 mRNA Vaccine Booster Shot: A Case Report. Front Med. (Lausanne).

[8] Rinaldi I., Pratama S., Wiyono L., Tandaju J.R., Wardhana I.L., Winston K. (2022). Efficacy and safety profile of COVID-19 mRNA vaccine in patients with hematological malignancies: Systematic review and meta-analysis. Front Oncol..

[9] Haggenburg S., Hofsink Q., Lissenberg-Witte B.I., Broers A.E.C., van Doesum J.A., van Binnendijk R.S., den Hartog G., Bhoekhan M.S., Haverkate N.J.E., Burger J.A. (2022). Antibody Response in Immunocompromised Patients With Hematologic Cancers Who Received a 3-Dose mRNA-1273 Vaccination Schedule for COVID-19. JAMA Oncol..

[10] Fromowitz A., Verma A. (2022). A needed boost against COVID-19 in lymphoma. Nat. Cancer.

[11] Ito Y., Honda A., Kurokawa M. (2022). COVID-19 mRNA Vaccine in Patients With Lymphoid Malignancy or Anti-CD20 Antibody Therapy: A Systematic Review and Meta-Analysis. Clin. Lymphoma Myeloma Leuk..

[12] Laracy J.C., Kamboj M., Vardhana S.A. (2022). Long and persistent COVID-19 in patients with hematologic malignancies: From bench to bedside. Curr. Opin. Infect. Dis..

